# Knockdown of USP8 inhibits prostate cancer cell growth, proliferation, and metastasis and promotes docetaxel’s activity by suppressing the NF-kB signaling pathway

**DOI:** 10.3389/fonc.2022.923270

**Published:** 2022-10-20

**Authors:** Md. Tariqul Islam, Fang-Zhi Chen, Han-Chun Chen, Abdul Wahid

**Affiliations:** ^1^ Department of Biochemistry and Molecular Biology, School of Life Sciences, Central South University, Changsha, China; ^2^ Department of Urology, The Second Xiangya Hospital of Central South University, Changsha, China; ^3^ Department of Cardiology of the Third Xiangya Hospital, Central South University, Changsha, Hunan, China

**Keywords:** prostate cancer, USP8, EGFR, PI3K, Docetaxel, NF-kB

## Abstract

Ubiquitin-specific protease 8 (USP8) has been recently reported to be involved in tumorigenesis. Prostate cancer (PCa) is the most diagnosed malignancy among men, but USP8’s role in PCa is not yet investigated comprehensively. Therefore, the PCa cell lines DU145 and PC3 were transfected with USP8 siRNA or overexpressing vector together with or without docetaxel. The silencing USP8 and docetaxel treatment reduced cell viability and migration and promoted apoptosis. In contrast, USP8 knockdown was found to enhance docetaxel antitumor activity. In contrast, increased cell viability and migration were noticed upon USP8 overexpression, thereby decreasing apoptosis and suppressing docetaxel antitumor activity. Notably, although EGFR, PI3K, and NF-kB were found to be increased in both USP8 overexpression and docetaxel treatment, it significantly attenuated the effects in USP8 silencing followed by with or without docetaxel. Although EGFR silencing decreased PI3K and NF-kB activation, overexpression of USP8 was shown to counteract SiEGFR’s effects on NF-kB signaling by increasing PI3K expression. Our findings revealed that USP8 plays an oncogenic role in PCa and can suppress docetaxel activity. Additionally, as EGFR/PI3K/NF-kB was previously reported to develop docetaxel resistance, the combination treatment of USP8 knockdown with docetaxel might be a potential PCa therapeutic.

## Introduction

Prostate cancer (PCa) is the most frequently diagnosed male malignancy and the second leading cause of cancer-related mortality worldwide. In the year 2021, globally an estimated 248,530 new cases of PCa were diagnosed (26% among the cancers) with approximately 34,130 deaths (11% among the cancers) (1). Androgen (AR) receptors play an essential role in prostate tumorigenesis; their activation promotes PCa cell growth while inhibiting apoptosis (2, 3). For advanced PCa, the standard first-line treatment is AR deprivation therapy (4). Clinical progress is seen when AR synthesis in the testes is reduced and/or AR receptor signaling is suppressed. Unfortunately, within 18–24 months, these individuals will regress and develop castration-resistant prostate cancer (CRPC) (5). CRPC patients have a terrible long-term prognosis, with a low overall survival (OS), albeit survival varies considerably based on individual disease features (6, 7).

Docetaxel has been on the market since 2004 and was reported to increase the OS of CRPC patients significantly (8). As a result, docetaxel-based chemotherapy is still considered the mainstay treatment for CRPC patients (9). However, docetaxel resistance is a substantial clinical issue. Tumors are either naturally docetaxel resistant or become docetaxel resistant, but in all cases, tumor growth goes away or returns quickly (8, 10). The development of docetaxel resistance in PCa has been researched in a variety of ways (10–13). Cancer drugs were reported to develop resistance through numerous deubiquitinating enzymes (DUBs) (14). The DUBs are involved in deubiquitination to remove the ubiquitin or ubiquitin chain from the substrate and stabilize it by protecting it from degradation and involvement in PCa tumorigenesis (15). The ubiquitin-specific proteases (USPs) are the most widespread DUB subfamily in humans. Ubiquitin-specific protease 8 (USP8), also known as UBPY, belongs to the USP family and is involved in protein endosomal sorting. USP8 is associated with tumor development, including lung cancer (16), cervical cancer (17), cholangiocarcinoma (18), breast cancer (19, 20), and hepatocellular carcinoma (21).

Downregulation of USP8 inhibits the survival of gefitinib-resistant non-small cell lung cancer (NSCLC) cells. It promotes apoptosis, advising that USP8 might be a therapeutic target for gefitinib-resistant NSCLC (16). However, it is yet unclear whether UPS8 is involved in the advancement of PCa. As a result, we looked into USP8’s biological function in PCa. The elevated EGFR, PI3K/Akt, and NF-kB activation was also previously reported to develop chemoresistance/drug resistance in numerous cancers (11, 22–24), whereas EGFR, PI3K/Akt, and NF-kB were previously reported to be regulated by ubiquitination and deubiquitination (25–28). However, the role of USP8 in PCa and whether USP8 has any effects on docetaxel treatment to regulate its effects targeting to enhance docetaxel activity, which may help to suppress the docetaxel resistance in CRPC, are also unclear. Therefore, we performed an *in vitro* study on DU145 and PC3 cell lines and observed the PCa cell’s growth, migration, apoptosis, and expression of EGFR, PI3K, and NF-kB-related proteins upon silencing or overexpressing USP8 followed by with or without docetaxel treatment.

Our results showed that USP8 has an oncogenic role in the proliferation and invasion of PCa cells, suggesting that it might be a novel therapeutic target in treating PCa. Furthermore, knocking down USP8 enhanced docetaxel’s anticancer activity. Also, it suppressed EGFR, PI3K, and NF-kB signaling pathways, whose elevated expression or activation was reported to develop docetaxel resistance in CRPC.

## Materials and methods

### Cell culture, transfection, and treatment

The DU145 and PC3 human PCa cell lines were purchased from the American Type Culture Collection (ATCC, USA). Both cell lines were cultured at 37°C, and 5% CO_2_ in RPMI 1640 medium (Gibco, MA, USA) was supplemented with 10% fetal bovine serum (Gibco) and 1% penicillin/streptomycin (Thermo Fisher Scientific, MA, USA). The cells were washed three times in PBS, digested with trypsin, and seeded into well plates for treatment and transfection after reaching the logarithmic growth phase. The MTT 3-(4,5-dimethylthiazol-2-Yl)-2,5-diphenyltetrazolium bromide assay was executed to calculate the IC_50_ of docetaxel for DU145 and PC3 cell lines.

pCMV3-USP8-Myc and pCMV3-C-Myc-Negative control vectors were obtained from Sino Biological Inc. (Beijing, China). USP8-SiRNA: 5′-GCU CGU AUU CAU GCA GAA ATT-3′, EGFR-SiRNA: 5′-GUA AUU AUG UGG UGA CAG ATT-3′, and negative control: 5′-UUC UCC GAA CGU GUC ACG UTT-3′, were obtained from GenePharma (Shanghai, China). USP8-pCMV3, control pCMV3, SiUSP8, SiEGFR, and control siRNA were transfected by Lipofectamine 2000 reagent (Invitrogen, MA, USA) as stated by the manufacturer.

### Assay for cell viability and proliferation

The MTT test was used to determine cell viability, with 10 μl of MTT reagent administered to each well of a 96-well plate (1 × 10^4^ cell/well). The absorbance at 490 nm was measured using a spectrophotometer. For MTT assay, PCa cell DU145 and PC3 were first transfected with SiUSP8 or USP8-pCMV3 along with their controls (scramble/mock) and incubated at different periods (0, 24, and 72 h). Later, the transfected cells (scramble/mock and SiUSP8/ovUSP8) were treated with or without docetaxel IC_50_ (4.5 nM in DU145 and 3 nM in PC3) and incubated for 48 h at 37°C and 5% CO_2_. The cell proliferation was measured by Trypan blue exclusion assay, and a hemocytometer was used to count the cells manually.

### Wound healing and Transwell assay

The DU145 and PC3 cells were cultured in six-well plates to carry out a wound-healing assay. After 24 h of incubation, the cells cultured in well plates were washed with PBS and a street wound line was created by pipette following the middle of each well between the cells. Then, the cells were transfected with USP8 siRNA or overexpression vector together with or without docetaxel IC_50_ treatment (4.5 nM in DU145 and 3 nM in PC3) and incubated at 37°C, and 5% CO_2_, and the images were taken under a fluorescence microscope (Olympus Corporation, Shinjuku, Japan) at different incubation periods like 0, 24, 48, and 72 h. For the Transwell migration test, the treated or transfected cells with controls were planted without serum in the top chamber (filter), while the lower chamber was filled with RPMI 1640 supplemented with 10% FBS. After allowing the cells to move for 48 h, they were treated with 0.1% crystal violet and dyed with it. A fluorescence microscope was used to obtain the images.

### Western blot analysis and antibodies

RIPA lysis buffer (Thermo Fisher Scientific) containing phenylmethylsulfonylfluoride (PMSF) (Solarbio, Beijing, China) and a protease inhibitor cocktail (Roche, Basel, Switzerland) was used to extract protein from the treated or transfected cells with controls. The BCA Protein Quantification Kit (Vazyme) was used to determine the protein content. Ten to 12% of SDS-PAGE was used to blot equivalent quantities of protein onto PVDF membranes. The blots were treated with primary antibodies overnight and then with a secondary antibody for 2 h the next day. Then, the blots were washed three times with TBST (Tris-buffered saline, 0.1% Tween 20), and blot images were taken by using a chemiluminescence kit. The following antibodies were used: USP8, EGFR, PI3K, P-Akt, IKKα, IκBα, GAPDH, E-cadherin, N-cadherin (Proteintech, IL, USA), p65, P-p65, P-IκBα, Cleaved Caspase 3, and Cleaved Caspase 9 (Wanleibio, Beijing, China).

### Flow cytometry for apoptosis analysis

In six-well culture plates with 2 ml per well, the DU145 and PC3 cells were seeded at 5 × 10^4^ cells/ml. Then, the cells were transfected with SiUSP8 or ovUSP8 with or without docetaxel IC_50_ (4.5 nM in DU145 and 3 nM in PC3) and incubated at 37°C with 5% CO_2_. After a 48-h incubation period, the cells were rinsed in ice-cold PBS, stained with 5 μl Annexin V/FITC (Beyotime, Beijing, China), and incubated in dark conditions for 5 min at room temperature, followed by 10 μl PI (propidium iodide, Beyotime) for 5 min. Cell apoptosis was detected using a flow cytometer, and the results were analyzed using the FlowJo software.

### Statistical analysis

The standard deviation (SD) of the mean of the group studies was calculated after each treatment or transfection was performed in triplicate. For each experiment to compare the groups, the Student’s two-tailed t-test was utilized, with p < 0.05 being statistically significant. One-way analysis of variance (ANOVA) and Fisher’s least significant difference tests were used to compare different groups, and p < 0.01 was considered statistically significant. GraphPad Prism 6 and IBM SPSS 17.0 were used to conduct these statistical analyses.

## Results

### USP8 regulates PCa cell growth and proliferation

**Figure 1:**
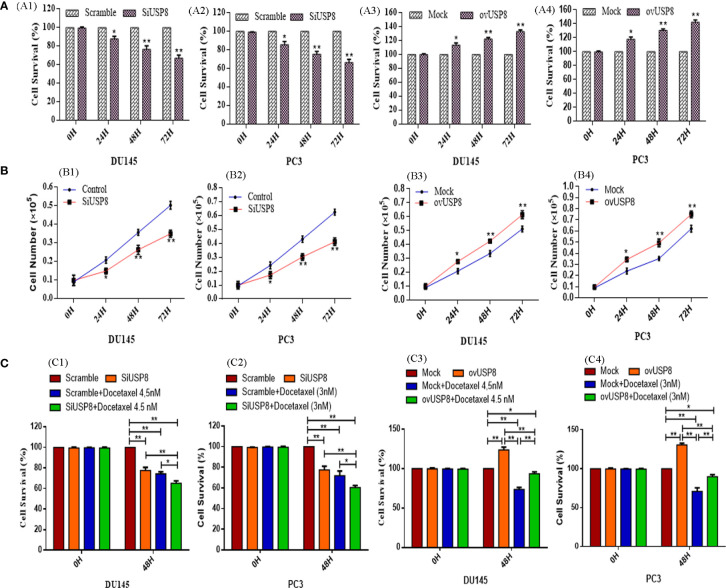
USP8 regulates PCa cell growth and proliferation. **(A)** DU145 and PC3 cell lines were transiently transfected with scramble siRNA, siUSP8, a mock vector, and a USP8-overexpressing vector. The MTT assay was used to determine cell survival at a different time point (0, 24, 48, 72 h). **(B)** Growth curves of DU145 and PC3 PCa cell lines. DU145 and PC3 PCa cells were transfected with scramble siRNA, siUSP8, a mock vector, and a USP8-overexpressing vector. Cells were cultured with a density of 1 × 10^5^ cells/ml. At the same time points, a total number of Trypan Blue-negative cells were manually counted and displayed. **(C)** DU145 and PC3 cells were transfected with scrambled siRNA or SiUSP8, and mock or USP8-overexpressing vector followed by docetaxel therapy with 4.5 and 3 nM, respectively. After 48 h, the cells were subjected to MTT assay and the cell survival from optical density (OD) calculated. The OD was set to 490 nm, and all data are presented as mean ± SD (n = 3, *p < 0.05 and **p < 0.01).

We used the MTT test to determine cell viability at various periods (0, 24, 48, and 72 h) by transfecting with SiUSP8. The cells transfected with scramble siRNA are considered as the control in each period of incubations. The optical density of scramble cells at each time point is considered as 100% cell survival. The viable percentages of PCa cells were nearly similar in 0 h of transfection. In contrast, significantly decreased viable cells were found in 24, 48, and 72 h upon SiUSP8 transfection compared to control in both PCa cell lines. For DU145, about 87.83 ± 2.74%, 76.6 ± 3.70%, and 67.1 ± 3.17% cells were monitored to survive upon SiUSP8 transfection over 24, 48, and 72 h, respectively (**Figure 1A1**). Additionally, for PC3, about 85.77 ± 3.4%, 75.6 ± 2.9%, and 66.47 ± 3.4% cells were monitored to survive upon SiUSP8 transfection over 24, 48, and 72 h, respectively (**Figure 1A2**).

In the trypan blue exclusion test, the growth or proliferation pattern of the control cells increased normally by increasing the cell numbers over time. In contrast, the silencing of USP8 showed a significantly decreased number of cells compared to control over 24, 48, and 72 h in both DU145 (**Figure 1B1**) and PC3 (**Figure 1B2**) cells, which also supports the MTT assay results.

Overexpression of USP8 improved the viability of PCa cells in a time-dependent way as compared to the mock-treated group. For DU145, about 113.67 ± 2.97%, 122.17 ± 2.4%, and 133.03 ± 2.31% cells were monitored to survive upon pCMV3-USP8 transfection over 24, 48, and 72 h, respectively (**Figure 1A3**). Additionally for PC3, about 117.77 ± 3.23%, 130.5 ± 2.2%, and 142.37 ± 3.05% of cells were monitored to survive upon pCMV3-USP8 transfection over 24, 48, and 72 h, respectively (**Figure 1A4**). The results of cell counting also backed up the MTT results. The number of cells in DU145 (**Figure 1B3**) and PC3 (**Figure 1B4**) upon overexpressing USP8 increased significantly throughout incubation at different periods. These results imply that USP8 plays a vital role in the development and proliferation of PCa cells, indicating that targeting USP8 for PCa treatment is a good idea.

The knockdown of USP8 enhanced the anti-proliferative impact of docetaxel in the MTT assay in both PCa cell lines. At 48 h of observation, both individual treatments of SiUSP8 and docetaxel were found to reduce survival in both PCa cells, which was found statistically significant compared to the control but did not find any significant difference in cell survival between SiUSP8 and docetaxel. However, docetaxel treatment was found to have lower cell survival (74.3 ± 2.1% in DU145 and 77.6 ± 3.6% in PC3) compared to SiUSP8 (77.67 ± 2.95% and 71.9 ± 4.5%) (**Figure 1C1**). Notably, the combination treatment of docetaxel and SiUSP8 was found to have the lowest cell survival 65.1 ± 2.4% and 60.4 ± 2.1% in DU145 (**Figure 1C1**) and PC3 (**Figure 1C2**) cells, respectively, which were significant compared to control and individual treatments of SiUSP8 and docetaxel.

On the other hand, compared to the control group, the USP8 overexpression eliminated the impact of docetaxel in PCa cells. The highest cell survivals 123.83 ± 3.43% and 130.5 ± 2.2% were noticed after 48 h of pCMV3-USP8 transfection in both DU145 (**Figure 1C3**) and PC3 (**Figure 1C4**) cells, respectively, which were significantly higher compared to control (100%), docetaxel treatment (73.63 ± 2.58% in DU145 and 70.93 ± 4.52% in PC3), and combined treatment of ovUSP8 and docetaxel (93.6 ± 2.2% in DU145 and 89.73 ± 2.64% in PC3). Although the PCa cell survival in docetaxel treatment was found at 73.63 ± 2.58% in DU145 (**Figure 1C3**) and 70.93 ± 4.52% in PC3 (**Figure 1C4**), it significantly increased to 93.6 ± 2.2% and 89.73 ± 2.64% when docetaxel was treated along with ovUSP8 in DU145 (**Figure 1C3**) and PC3 (**Figure 1C4**) cells, respectively. According to the findings, USP8 has a function in the enhanced development and proliferation of PCa. It also proposes that USP8 may play a role in mediating docetaxel’s anticancer effects.

### Silencing of USP8 suppresses PCa cell migration and promotes docetaxel activity

**Figure 2:**
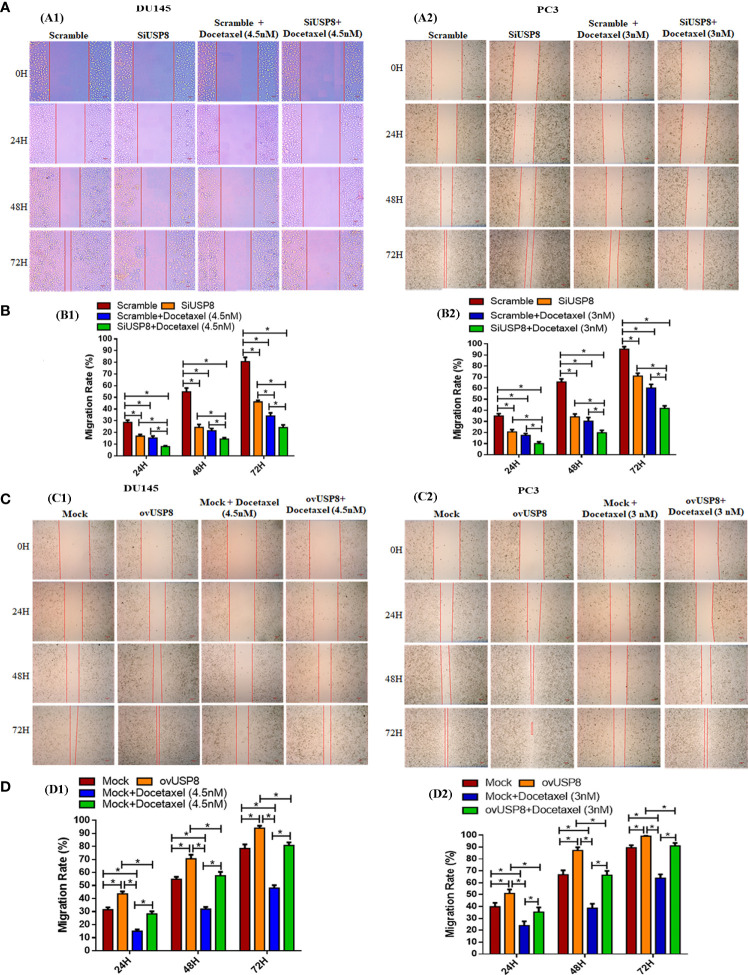
Regulation of PCa cell migration by USP8, and wound healing action of docetaxel. **(A)** PCa cells DU145 and PC3 were transfected with either scrambled or SiUSP8, and wound healing assay was employed followed by with or without docetaxel therapy (4.5 nM in DU145 and 3 nM in PC3) as described details in the methodology section. **(B)** The un-migrated area was measured by using ImageJ, and bar diagrams were plotted to represent the rate of cell migration of each independent experiment (n = 3, *p < 0.05). **(C)** PCa cells DU145 and PC3 were transfected with either mock vector or pCMV3-USP8-overexpressing vector, and wound healing assay was employed in the presence or absence of docetaxel (4.5 nM in DU145 and 3 nM in Pc3) as described details in methodology. **(D)** The un-migrated area was measured by using ImageJ, and bar diagrams were plotted to represent the rate of cell migration of each independent experiment (n = 3, *p < 0.05).

In DU145, silencing of USP8 was found to have significantly reduced cell migration rates 16.8 ± 1.5%, 24.3 ± 2.6%, and 46.2 ± 1.4% compared to control with 28.6 ± 1.9%, 54.8 ± 3.2%, and 80.6 ± 3.7% at 24, 48, and 48 h, respectively (**Figures 2A1, B1**). The docetaxel treatment in scramble DU145 cells found 15.3 ± 1.6%, 21.5 ± 1.9%, and 34.1 ± 2.7% of migrated area, which was also significantly lower than the control at 24, 48, and 72 h, respectively (**Figures 2A1, B1**). However, the percentage of cell-migrated areas in docetaxel-treated DU145 was found lower compared to USP8 knockdown at a different point of time. Still, it was only significant at 72 h of observation (34.1 ± 2.7% in docetaxel and 46.2 ± 1.4% in SiUSP8) (**Figures 2A1, B1**). The lowest migration rates of 7.9 ± 0.8%, 14.5 ± 1.2%, and 24.2 ± 2.3% were found in docetaxel-treated silenced USP8 cells of DU145 at 24, 48, and 72 h, respectively, which was significant not only compared to the control but also compared to the individual treatments of SiUSP8 and docetaxel (**Figures 2A1, B1**).

Similarly, in PC3, silencing of USP8 was found to have significantly reduced cell migration rates at 20.6 ± 2.0%, 34.1 ± 2.7%, and 71.0 ± 2.5% compared to control at 34.9 ± 2.1%, 65.7 ± 2.6%, and 95.3 ± 2.3% over 24, 48, and 48 h, respectively (**Figures 2A2, B2**). The docetaxel treatment in scramble PC3 cells found 17.5 ± 1.7%, 30.3 ± 3.2%, and 60.2 ± 3.4% migrated areas, which was also significantly lower than the control at 24, 48, and 72 h, respectively (**Figures 2A2, B2**). Although the percentage of cell-migrated areas in docetaxel-treated PC3 was found lower at a different time compared to USP8 knockdown, the differences were not statistically significant. The lowest migration rates 9.9 ± 1.7%, 19.8 ± 2.3%, and 41.8 ± 2.3% were found in docetaxel-treated silenced USP8 cells of PC3 at 24, 48, and 72, respectively, which was significant not only compared to the control but also compared to individual treatments of SiUSP8 and docetaxel (**Figures 2A2, B2**). By these findings, USP8 has a significant role in PCa cell migration. Its knockdown inhibits the cell migration and enhances the healing action of docetaxel in both cells.

**Figure 3:**
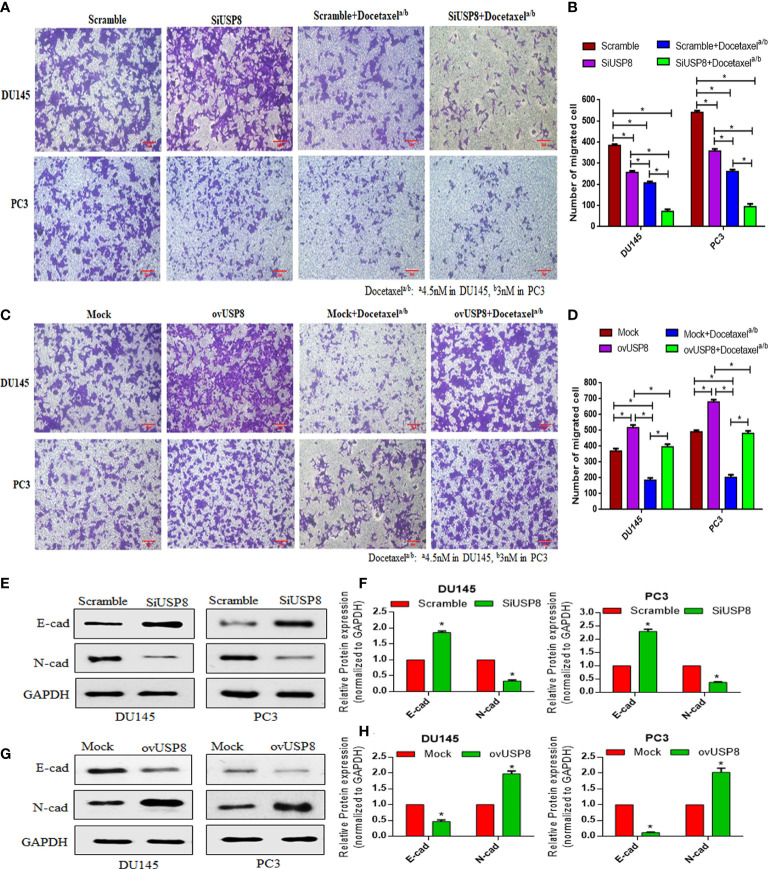
Regulation of the number of PCa migrating cells by USP8, and the anti-migrating action of docetaxel in Transwell assay through regulation of EMT-related proteins. **(A)** Scrambled or USP8-specific siRNA was transfected into PCa cells. Docetaxel was used to treat transfected cells before they were introduced to the Transwell chamber. The migrating cells were treated with 4% paraformaldehyde before being imaged (100×). **(B)** Mock or pCMV3-USP8-overexpressing vector was transfected into PCa cells. Docetaxel was used to treat transfected cells before they were introduced to the Transwell chamber. The migrating cells were treated with 4% paraformaldehyde before being imaged (100×). **(C, D)** ImageJ software was used to determine the number of migratory cells, which was shown on a graph. Data are represented as mean ± SD (n = 3, *p < 0.05). The DU145 and PC3 cells were transfected with SiUSP8 **(E)** and pCMV3-USP8-overexpressing vector **(F)** for 48 h; total protein was extracted and incubated with antibody (E-cadherin and N-cadherin) after executing Western blotting. GAPDH was utilized as a control in Western blotting. **(G, H)** The relative gray value was measured using ImageJ software and plotted on a bar diagram by GraphPad prism. The data were standardized using GAPDH as a loading control and given as mean ± SD (n = 3, *p < 0.05).

After that, cell migration was investigated using a Transwell migration test to validate the findings. Compared to the control group, suppression of USP8 resulted in a lower chamber with a significantly smaller number of migrating cells but a considerably higher number of migrating cells were found compared to docetaxel treatment (**Figure 3A**). The lowest amount of migrating cells observed in the combined therapy of docetaxel and SiUSP8, which showed significance compared to all other investigating groups in both DU145 and PC3 cells (**Figures 3A, C**). Therefore, the migration results in the Transwell were similar to the wound healing assay and supporting consequences for both PCa cell lines.

### Overexpression of USP8 promotes PCa cell migration and diminished docetaxel activity

In DU145, the highest cell migration rates were 43.6 ± 1.8%, 70.5 ± 3.1%, and 94.04 ± 1.8% at 24, 48, and 72 h of observation, respectively, upon pCMV3-USP8 transfection, which was significant not only compared to mock control at 31.5 ± 1.8%, 54.9 ± 1.8%, and 78.5 ± 3.1% over 24, 48, and 48 h, respectively, but also compared to docetaxel treatment and docetaxel treatment with pCMV3-USP8 transfection in different periods of observations (**Figures 2C1, D1**). The docetaxel treatment in mock DU145 cells found 15.04 ± 1.3%, 31.9 ± 1.6%, and 48.03 ± 2.3% migrated areas at 24, 48, and 72 h, respectively, which were found to be significantly lower than those of the control and ovUSP8 (**Figures 2C1, D1**). Although the migration rate in docetaxel-treated DU145 was found the most deficient in every period of observation, the migration rates were significantly increased at 28.2 ± 1.9%, 57.6 ± 2.9%, and 80.8 ± 2.3% for 24, 48, and 72 h of observation, respectively, when docetaxel was treated in USP8-overexpressed cells. In addition, pCMV3-USP8 and docetaxel showed a significantly lower migration rate than only pCMV3-USP8-transfected DU145 cells in different periods of observation. Still, pCMV3-USP8 and docetaxel did not show any significant difference compared to the mock control (**Figures 2C1, D1**).

Similarly, in PC3, the highest cell migration rates were 51.1 ± 3.4%, 87.2 ± 2.7%, and 99.2 ± 0.3% at 24, 48, and 72 h of observation, respectively, upon pCMV3-USP8 transfection, which was significant not only compared to mock control 39.9 ± 3.3%, 66.8 ± 3.7%, and 89.5 ± 2.1% at 24, 48, and 48 h, respectively, but also compared to docetaxel treatment and docetaxel treatment with pCMV3-USP8 transfection in different periods of observations (**Figures 2C2, D2**). The docetaxel treatment in mock PC3 cells found 23.9 ± 3.7%, 38.7 ± 3.6%, and 63.9 ± 3.1% migrated areas, which was also significantly lower than control and ovUSP8 at 24, 48, and 72 h, respectively (**Figures 2C2, D2**). Although the migration rate in docetaxel-treated PC3 was found the lowest in every period of observation, the migration rate was significantly increased when docetaxel was treated in USP8-overexpressed cells with 35.4 ± 3.9%, 66.4 ± 3.7%, and 91.0 ± 2.5% for 24, 48, and 72 h of observation, respectively. In addition, pCMV3-USP8 along with docetaxel showed a significantly lower migration rate compared to only pCMV3-USP8-transfected PC3 cells in different periods of observation. Still, the pCMV3-USP8 and docetaxel did not show any significant difference compared to mock control (**Figures 2C2, D2**). By these findings, USP8 has a significant role in PCa cell migration. Its overexpression increased the PCa cell migration and diminished the healing action of docetaxel in both cells.

Similar to the wound healing assay, overexpression of USP8 resulted in the highest number of migrated cells for both Du145 and PC3 cells in the Transwell assay, which showed statistical significance compared to all other treatment group cells (**Figure 3B**). On the other hand, although the lowest number of migrated cells was noticed in docetaxel treatment, it was significantly increased in the combined therapy of docetaxel and pCMV3-USP8-overexpressing vector cells (**Figures 3B, D**). Therefore, similar to the wound healing assay, it could be stated that USP8 overexpression increased the PCa cell migration and diminished the effect of docetaxel on PCa migration.

### USP8 regulates EMT-related proteins leading to PCa cell migration and metastasis

A significantly increased expression of epithelial cadherin (E-cadherin) was noticed upon USP8-specific siRNA transfection. In contrast, the expression of neural cadherin (N-cadherin) was significantly decreased in both PCa cell line DU145 cells and PC3 cells (**Figures 3E, G**). On the other hand, significantly reduced E-cadherin and elevated N-cadherin were found upon USP8 overexpression in both PCa cell line cells (**Figures 3F, H**). The results show that USP8 can promote the epithelial-to-mesenchymal transition (EMT) process by decreasing E-cadherin and increasing N-cadherin, thereby increasing the PCa cell migration and metastasis.

### USP8 silencing downregulates the NF-κB pathway and inhibits docetaxel-mediated NF-κB activation

**Figure 4:**
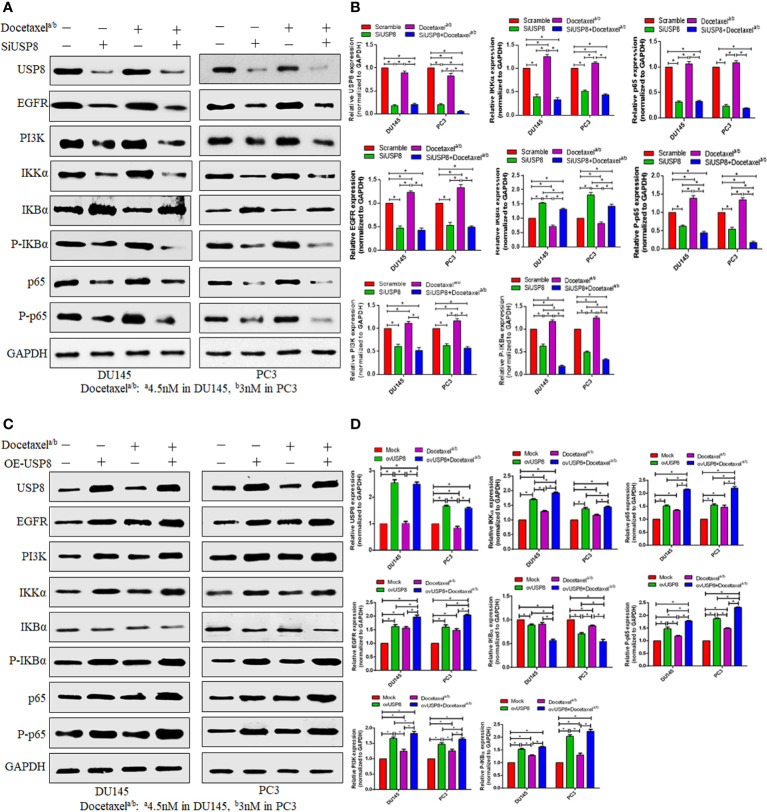
USP8 regulates the NF-κB pathway and docetaxel-mediated NF-κB activation in PCa. **(A)** The DU145 and PC3 cells were transfected or treated with siUSP8 and docetaxel IC_50_ individually or combined, and for Western blotting, complete protein was obtained. Antibodies to the indicated proteins (USP8, EGFR, PI3K, IKKα, IKBα, P-IKBα, p65, P-p65) were used to detect their expression. As a control, GAPDH was employed. **(B)** ImageJ was used to compute the relative gray value of the blots. The results were standardized using GAPDH as a loading control (individual protein/GAPDH) and shown as mean ± SD from three replicated experiments (n = 3, *p < 0.05). **(C)** The DU145 and PC3 cells were treated with USP8-overexpressing plasmid and docetaxel IC_50_ individually or combinedly, and for Western blotting, complete protein was obtained. Antibodies to the indicated proteins (USP8, EGFR, PI3K, IKKα, IKBα, P-IKBα, p65, P-p65) were used to detect their expression. As a control, GAPDH was employed. **(D)** ImageJ was used to compute the relative gray value of the blots. The results were standardized using GAPDH as a loading control (individual protein/GAPDH) and shown as mean ± SD from three replicated experiments (n = 3, *p < 0.05).

To find whether USP8 and docetaxel could regulate the NF-κB signaling pathway by an individual or combined treatment in PCa, we extracted the total protein. We performed Western blotting of DU145 and PC3 cell lines upon transfection of siUSP8 with or without docetaxel treatment. The USP8 silencing in both PCa cell lines found a significantly decreased IKKα and thereby decreased phosphorylated IκBα and increased IκBα compared to control and docetaxel treatment (**Figures 4A, B**). Furthermore, both phosphorylated and unphosphorylated p65 were found to be decreased significantly upon USP8 knockdown compared to control and docetaxel treatment. Interestingly, the NF-κB upstream proteins EGFR and PI3K were also found to be reduced significantly by siRNA-mediated silencing of USP8 compared to control and docetaxel treatment (**Figures 4A, B**).

On the other hand, the docetaxel treatment activated the NF-κB signaling pathway by increasing IKKα and phosphorylated IκBα and p65. A significantly decreased IκBα was found in the docetaxel treatment, but no significant difference in p65 was found in the docetaxel treatment compared to the control. Additionally, significantly increased expressions of EGFR and PI3K were also found in docetaxel treatment compared to control (**Figures 4A, B**). Interestingly, although the docetaxel treatment increased EGFR and PI3K and the NF-κB signaling pathway, the lowest EGFR, PI3K, and decreased NF-κB signaling pathways were found when the USP8-silenced PCa cells were treated with docetaxel. The significant proteins involved in activating NF-κB signaling pathways, such as IKKα and phosphorylated IκBα and p65, were found the lowest in the combination treatment of siUSP8 and docetaxel in both PCa cell lines (**Figures 4A, B**).

### USP8 overexpression upregulates the NF-κB pathway and enhances docetaxel-mediated NF-κB activation

It was previously reported that docetaxel enhanced NF-κB activation, which was attenuated by silencing USP8. Later, we inquired into the possibility of overexpression of USP8 in the NF-κB activation and its involvement in docetaxel-mediated NF-κB activation. We performed vector-mediated overexpression of USP8 together with or without docetaxel treatment in the DU145 and PC3 cell lines. The data from Western blotting showed that there was a significantly increased IKKα, phosphorylated IκBα and p65, and p65 found in USP8-overexpressed PCa cells but decreased IκBα compared to control and thereby upregulating the NF-κB activation (**Figures 4C, D**). Consequently, significantly increased EGFR and PI3K were also found in USP8-overexpressed PCa cell lines compared to the control. Similar results were also found in docetaxel treatment where PI3K, phosphorylated IκBα, and p65 were significantly lower than USP8-overexpressing PCa cells. Therefore, both the USP8 overexpression and docetaxel treatment were individually found to enhance NF-κB activation, where their combination treatment found the highest level of EGFR, PI3K, IKKα, phosphorylated IκBα and p65, and p65 and the lowest IκBα and thereby severely enhancing NF-κB activation in PCa cells (**Figures 4C, D**).

### USP8 regulates apoptosis and docetaxel-mediated apoptosis in prostate cancer

**Figure 5:**
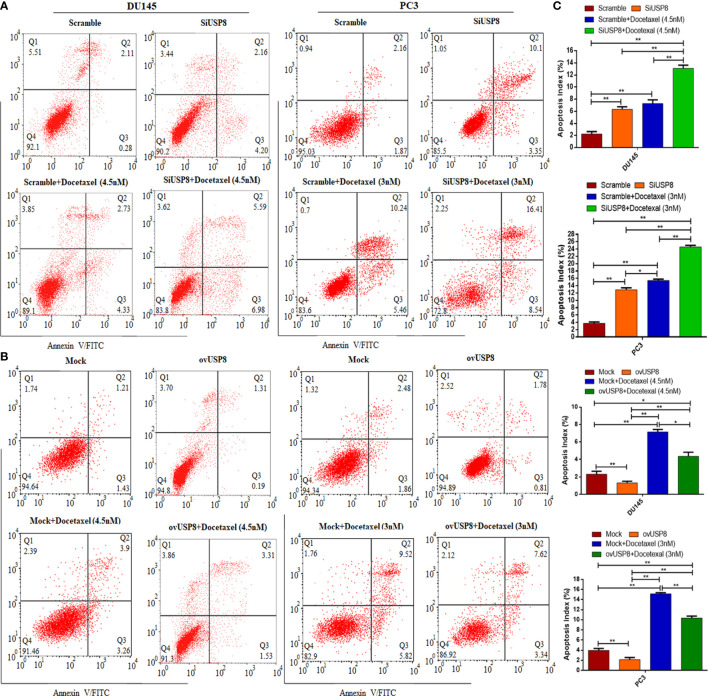
Knockdown of USP8 increases apoptosis and enhances docetaxel-mediated apoptosis in PCa where its overexpression shows the opposite effects. **(A)** The DU145 and PC3 cells were transfected or treated with siUSP8 and docetaxel IC_50_ individually or combined, and the apoptosis was examined using a flow cytometry assay. **(B)** The DU145 and PC3 cells were transfected or treated with pCMV3-USP8 and docetaxel IC_50_ individually or combined, and the apoptosis was examined using a flow cytometry assay. The four-quadrant pictures observed by flow cytometric analysis are shown by the dot plot. Q1 shows necrotic cells, Q2 shows later period apoptotic cells, Q3 shows early apoptotic cells, and Q4 shows normal cells. **(C)** The total percentage of apoptotic cells (sum of apoptotic cells and early apoptotic cells) shown in the bar diagram to compare the value between and among the study groups. The data are shown as mean ± SD from three replicated experiments (n = 3, **p < 0.01; *p <0.05).

The data in **Figure 5A** indicated that silencing of USP8 significantly increased the apoptotic cells compared to control (control vs. siUSP8: DU145, 2.26 ± 0.38% vs. 6.32 ± 0.44%, P value = 0.0003; PC3, 3.73 ± 0.36% vs. 12.89 ± 0.5%, P value ≤0.0001). The apoptotic cells were also found to be significantly increased in docetaxel treatment compared to control (control vs. docetaxel: DU145, 2.26 ± 0.38% vs. 7.29 ± 0.59%, P value = 0.0002; PC3, 3.73 ± 0.36% vs. 15.4 ± 0.4%, P value ≤0.0001). Interestingly, the docetaxel treatment found higher apoptotic cells compared to USP8 silencing in both PCa cell lines, which was significant in PC3 (siUSP8 vs. docetaxel: 12.89 ± 0.5% vs. 15.4 ± 0.4%, P value = 0.003) but not in DU145 (siUSP8 vs. docetaxel: 6.32 ± 0.44% vs. 7.29 ± 0.59%, P value = 0.2). More importantly, the highest percentage of apoptotic cells 13.1 ± 0.5% and 24.6 ± 0.4% were found in DU145 and PC3 cell lines, respectively, which were significantly higher compared to control, siUSP8, and docetaxel treatment in both PCa cell lines (P value ≤0.0001 for all comparisons, **Figure 5C**).

The data in **Figure 5B** showed that overexpression of USP8 significantly decreased the apoptotic cells compared to control (control vs. ovUSP8: DU145, 2.3 ± 0.34% vs. 1.3 ± 0.2%, P value = 0.007; PC3, 3.9 ± 0.38% vs. 2.1 ± 0.4%, P value ≤0.001), whereas the apoptotic cells were found to be significantly increased in docetaxel treatment compared to control (control vs. docetaxel: DU145, 2.3 ± 0.34% vs. 7.15 ± 0.28%, P value = 0.0013; PC3, 3.9 ± 0.38% vs. 15.11 ± 0.24%, P value ≤0.0001). Additionally, the docetaxel treatment found significantly higher apoptotic cells compared to USP8 overexpression in both PCa cell lines (ovUSP8 vs. docetaxel: DU145, 1.3 ± 0.2% vs. 7.15 ± 0.28%, P value = 0.0006; PC3, 2.1 ± 0.4% vs. 15.11 ± 0.24%, P value ≤0.0001). Interestingly, this apoptotic role of docetaxel was found to be attenuated upon USP8 overexpression. The percentages of increased apoptotic cells in docetaxel treatment were found to decrease significantly in the combination treatment of USP8 overexpression with docetaxel (docetaxel vs. ovUSP8+Docetaxel: DU145, 7.15 ± 0.28% vs. 4.36 ± 0.41%, P value = 0.016; PC3, 15.11 ± 0.24% vs. 10.35 ± 0.37%, P value ≤0.002). Moreover, although the ovUSP8+Docetaxel treatment found significantly lower apoptotic cells (%) compared to the docetaxel treatment, it found significantly higher apoptotic cells (%) compared to control and USP8 overexpression in both PCa cell lines (**Figure 5B, C**).

**Figure 6:**
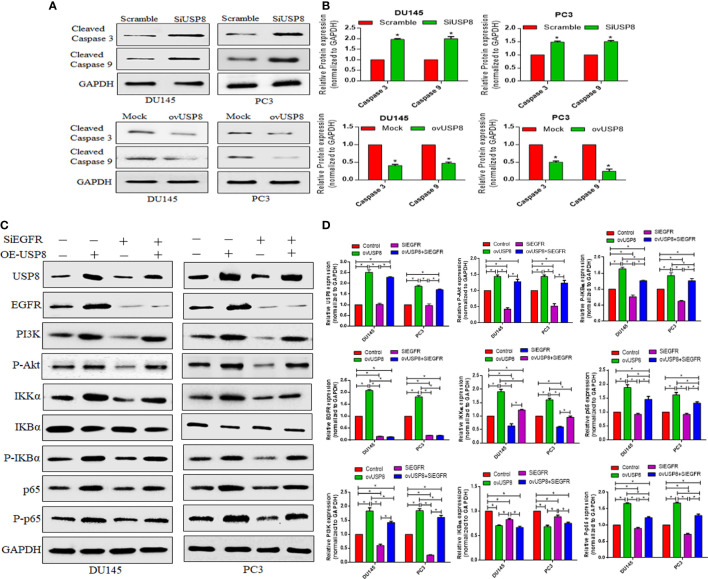
USP8 regulates pro-apoptotic proteins and upregulates the NF-κB pathway through EGFR and PI3K in PCa cells. **(A)** The DU145 and PC3 cells were transfected with SiUSP8 and pCMV3-USP8-overexpressing vector for 48 h. Western blotting was conducted after total protein extraction. The indicated antibodies (cleaved caspase 3 and cleaved caspase 9) were used to observe their expression. The GAPDH was utilized as a control in Western blotting. **(B)** The relative gray value was measured using ImageJ software and plotted on a bar diagram by GraphPad Prism. The data were standardized using GAPDH as a loading control and given as mean ± SD (n = 3, *p <0.05). **(C)** The DU145 and PC3 cells were treated with USP8 overexpressing plasmid and SiEGFR individually or combinedly, and for Western blotting, complete protein was obtained. Antibodies to the indicated proteins (USP8, EGFR, PI3K, IKKα, IKBα, P-IKBα, p65, P-p65) were used to detect their expression. As a control, GAPDH was employed. **(D)** ImageJ was used to compute the relative gray value of the blots. The results were standardized using GAPDH as a loading control (individual protein/GAPDH) and shown as mean ± SD from three replicated experiments (n = 3, *p < 0.05).

To further understand the mechanism of USP8 behind the increased apoptosis, a Western blot was used to investigate the expression of apoptosis-related proteins. So, for this, we examined the expression of cleaved Caspase 3 and cleaved Caspase 9 pro-apoptotic proteins upon USP8 siRNA and pCMV3-USP8-overexpressing vector transfection in both DU145 and PC3 cells. In all PCa cell lines, in comparison to the control group, knocking down USP8 increased cleaved Caspase 3 and cleaved Caspase 9 levels (**Figures 6A, B**). In contrast, the elevated expression of USP8 greatly reduced the proportion of apoptotic PCa cells (**Figure 5B**) and reduced the cleaved Caspase 3 and cleaved Caspase 9 which were found in DU145 and PC3 cells by USP8 overexpression (**Figures 6A, B**). These findings propose that USP8 may regulate the Caspase cascade’s apoptosis in PCa cells.

### USP8 regulates NF-κB activation through EGFR and PI3K stabilization to promote PCa cell proliferation and survival

In multiple studies (29–32), the EGFR is a substrate of USP8 deubiquitination, while USP8 has been shown to enhance gastric cancer cell proliferation and metastasis *via* the PI3K/AKT signaling pathway (30). This current study showed that the EGFR, PI3K, and NF-κB signaling is downregulated and upregulated in PCa by USP8 silencing and overexpressing, respectively (**Figure 4**). Moreover, PI3K/Akt is a well-established downstream target of EGFR, which is generally activated by EGFR autophosphorylation leading to cell proliferation and growth (33–35). However, the underlining mechanism of USP8 in regulating EGFR and PI3K targeting to activate the NF-κB signaling pathway in PCa is not clearly stated yet. Therefore, this study also examined the effects of overexpressed USP8 and silenced EGFR in regulating NF-kB activation through stabilizing EGFR and PI3K.

pCMV3-USP8 significantly increased USP8 expression, whereas no changes in the USP8 expression by knockdown of EGFR were found. The highest levels of EGFR and PI3K were found upon USP8 overexpression in both PCa cell lines, which was statistically significant compared to all other study groups (**Figure 6C, D**). Similarly, the highest P-Akt was found upon USP8 overexpression in both PCa cell lines, which was also statistically significant compared to control and SiEGFR transfection but not to ovUSP8+SiEGFR treatment. Although the EGFR expression was found to be significantly lower in siRNA-mediated EGFR silencing and as well as in ovUSP8+SiEGFR treatment compared to control, the significantly reduced PI3K and P-Akt were only found in silenced EGFR cells, but interestingly, their expression was elevated in ovUSP8+SiEGFR treatment. Therefore, the elevated level of PI3K and P-Akt was found in both ovUSP8 and ovUSP8+SiEGFR treatment, whereas their expression was lower in the control group and SiEGFR treatment. Thereby, together with these data, it can be concluded that the elevated level of USP8 not only increases the expression of EGFR, PI3K, and P-Akt but also might increase the PI3K and P-Akt expression over EGFR silencing where the PI3K and P-Akt well established the downstream target of EGFR (**Figures 6C, D**).

Similar to EGFR, PI3K, and P-Akt, the highest levels of IKKα, P-IKBα, p65, and P-p65 were found upon USP8 overexpression. In contrast, their lowest expression was noticed upon EGFR silencing in DU145 and PC3 cells. The reduced IKKα, P-IKBα, p65, and P-p65 in EGFR silenced cells were found to increase significantly when the EGFR-deficit PCa cells were treated by pCMV3-USP8 (ovUSP8+SiEGFR). Additionally, IKBα was significantly lower in both ovUSP8 and ovUSP8+SiEGFR treatment compared to the control and EGFR silencing groups (**Figures 6C, D**). Therefore, by these findings, it can be concluded that USP8, EGFR, PI3K, and P-Akt can activate NF-κB signaling where USP8 is a regulator of EGFR, PI3K, and P-Akt in PCa cell proliferation and survival.

## Discussion

USP8, a member of the largest deubiquitinase (USP) family, has been associated with the development of a variety of cancers, including breast cancer (19, 20), lung cancer (16, 36), and cervical squamous cell carcinoma (17), as previously stated. USP8 has been found to perform an oncogenic function in lung cancer and cervical squamous cell carcinoma, with high expression associated with a poor prognosis (17, 36); however, USP8 has also been found to predict improved survival in breast cancer patients (37); these data show that USP8 plays various functions in different malignancies.

This is the first time we showed that knocking down USP8 inhibited PCa cell proliferation, migration, and invasion decreasing the NF-κB signal through the suppression of EGFR and PI3K. In contrast, overexpression of USP8 resulted in strong proliferative effects, suggesting that USP8 functions as an oncogene in prostate cancer growth, survival, and metastasis. Besides, we also showed that knocking down of USP8 in PCa cells enhanced the anticancer activity of docetaxel whereas the overexpression of USP8 suppressed the docetaxel activity. Reduced E-cadherin and increased N-cadherin promote the EMT process for cancer cell migration and metastasis (38–40). Here in this study, we found that knocking down USP8 significantly inhibited the PCa cell migration by suppressing the EMT process through the increased E-cadherin and decreased N-cadherin, whereas USP8 overexpression promoted the EMT process through the decreased E-cadherin and increased N-cadherin and so thereby increased the migration of both DU145 and PC3 cells.

Knocking down USP8 downregulated the NF-κB signal by decreasing its intermediatory proteins (IKK, P-IKB, p65, and P-p65), whereas USP8 overexpression promoted the NF-κB signal by increasing its intermediatory proteins. On the other hand, the docetaxel treatment was found to promote the NF-κB signal in both DU145 and PC3 cells. Interestingly, the silencing of USP8 and docetaxel treatment attenuated the docetaxel-mediated NF-κB signal activation. In addition, the activation of the NF-κB signaling pathway was found in PCa (41) and was reported to develop docetaxel resistance in CRPC (42). Not only this, although the docetaxel treatment reduces EGFR activation by inhibiting its being phosphorylated in the presence of EGF (43), the abnormal activation, mutation, and overexpression of EGFR played a vital role in the establishment of docetaxel resistance in tumor cells as well as PCa cells (11, 44–46). In this present study, we found increased EGFR, PI3K, and NF-κB signal upon USP8 overexpression and docetaxel treatment in DU145 and PC3 cells, and knockdown of USP8 found reduced EGFR, PI3K, and NF-κB signal. Interestingly, the docetaxel-mediated upregulation of EGFR, PI3K, and NF-κB signal decreased by USP8 silencing.

It has been shown that USP8 can control the stability of EGFR *via* modulating intracellular transit and recycling by deubiquitination and is found overexpressed and correlated in multiple cancers (16, 25, 29, 30, 45). Byun et al. reported the USP8 silencing as a novel target to overcome gefitinib-mediated resistance in lung cancer, where gefitinib is a potent inhibitor of EGF receptor tyrosine kinase (EGFR-TKIs) (16). The previous study reported that the EGFR increase, EGFR-mediated PI3K/Akt activation, and increased NF-κB signal activation were found to develop docetaxel resistance in CRPC (11, 12, 42), whereas this present study found reduced EGFR, PI3K, and NF-κB signal by silencing USP8 in with or without docetaxel treatment (**Figure 4A**). Therefore, the inhibition of USP8 might be a novel therapeutic target to inhibit PCa cell growth, proliferation, and metastasis and overcome docetaxel-mediated resistance in CRPC by suppressing EGFR, PI3K, and NF-kB signals.

One of the critical characteristics of tumor cells is their ability to avoid programmed death, known as apoptosis. As a result, encouraging tumor cell death might be an efficient way to slow tumor development. USP8 inhibited extrinsic apoptosis in HeLa cells through the long isoform of FLICE-like inhibitory protein (FLIP_L_) deubiquitylation and stabilization, where FLIP_L_ is a critical apoptosis regulator mediated by the death receptor (47). This current study also found significantly increased apoptotic PCa cells upon silencing USP8 compared to control, whereas its overexpression significantly reduced the apoptotic cells compared to control. Although the docetaxel-mediated apoptosis in PCa significantly increased when the USP8-silenced PCa cells were treated with docetaxel, the USP8 overexpression significantly suppressed the docetaxel-mediated apoptosis in both DU145 and PC3 cells. Therefore, USP8 has an anti-apoptotic activity in PCa over docetaxel treatment. Previously, it was reported that knocking down USP8 promotes apoptosis by downregulating FLIP_L_ and upregulating cleaved Caspase 3 and cleaved Caspase 8 in HeLa cells (47). Jing et al. extended the apoptotic activity of USP8 in cholangiocarcinoma cells, where the silencing of USP8 was reported to decrease Bcl-2 expression and increase Bax, cleaved Caspase 3, and cleaved Caspase 9 expression and thereby triggering apoptosis (18).

Here, we also found significantly increased cleaved Caspase 3 and cleaved Caspase 9 by silencing USP8 whereas the opposite was found by USP8 overexpression in both DU145 and PC3 cells. Bcl-2/Bax and Caspase 9 are well-known regulators of the intrinsic apoptosis pathway, with Caspase 3 serving as the primary executor of a sequence of apoptotic actions (48). Although in HeLa cells silencing USP8 promotes death receptor-mediated extrinsic apoptosis (47), here we found that USP8 affects apoptosis in PCa *via* modulating the intrinsic apoptosis pathway, and its influence on extrinsic apoptosis in PCa is needed to be investigated more in the future.

**Figure 7:**
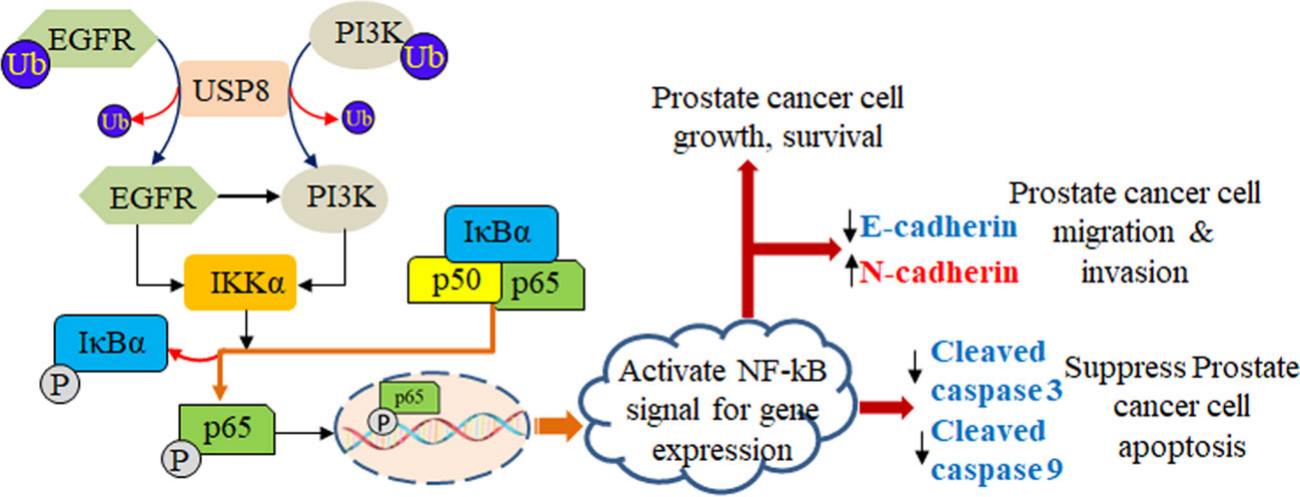
Role of USP8 in prostate cancer. USP8 stabilizes both EGFR and PI3K, which leads to elevation in the expression of IKKα. The elevated IKKα phosphorylates IkBα and dissociates p65 from IkBα and is translocated into the nucleus acting as a transcription factor that activates the NF-kB signaling pathway, thereby increasing prostate cancer cell growth and survival as well as prostate cancer cell migration and invasion by increasing N-cadherin and decreasing E-cadherin, and suppressing prostate cancer cell apoptosis by decreasing cleaved caspase 3 and cleaved caspase 9.

EGFR has been studied as a potential target in various solid cancers, including lung, bladder, colon, breast, and head and neck carcinomas. These studies’ findings imply that drugs targeting EGFR may be helpful in slowing the development of some solid cancers (49). The EGFR overexpression and hormone-refractory actions were previously shown to be linked in PCa to progress the cells more aggressively (50). As a result, inhibition of increased EGFR expression may consider a potential therapeutic target; if not, then it might be challenging to treat a subset of PCa (51). Similar to Marks et al., we found an elevated EGFR expression in DU145 and PC3 which was severely increased upon USP8 overexpression and docetaxel treatment, whereas silencing USP8 significantly reduced EGFR expression and thereby decreased NF-kB signal activation. We also used EGFR-specific siRNA and found decreased NF-kB signal activation considerably. Still, the overexpression of USP8 significantly attuned to the effects of EGFR silencing in PCa. This might be due to the increased level of PI3K and P-Akt by USP8 overexpression. Although the decreased PI3K and P-Akt levels were found by silenced EGFR, the overexpressed USP8 was shown to increase PI3K/P-Akt expression over EGFR silencing and thereby increase the NF-kB signal activation (**Figure 7**).

The link between dysfunctional PI3K/Akt activity and cancer development is widely established. PI3K was reported as the most often activated growth factor pathway in androgen-dependent LNCaP PCa cells (52). Lung cancer (53), leukemia (54), breast cancer (55), and melanomas (56) were found in the direct implication of PI3K activity for tumor progression. Additional data support that Akt, a downstream kinase of PI3K, is also responsible for cell malignancy and hormone resistance development (57). Activated Akt can interfere with the production and control of hormone receptor reactions, making hormone ablation therapy ineffective (58).

Several studies have shown that EGFR overexpression or unregulated activation is linked to PCa development *in vivo* (59). It was also reported that the EGFR has been linked to developing resistance against anticancer drugs (60, 61). The increased expression of EGFR in cancer cells was a significant factor in the development of chemo-resistance (44, 45). The action of EGFR was found to increase significantly in both human primary PCa cells and CRPC cells (62). Our data showed that expressions of EGFR, PI3K, and P-Akt were significantly higher in DU145 and PC3 cells and docetaxel treatment increased them severely to upregulate the NF-kB signal. In contrast, the USP8-specific siRNA reduced EGFR and PI3K, suppressing the NF-kB signal. The disruption of EGFR by inhibiting UP8 was reported to overcome gefitinib resistance in lung cancer (16). Our findings showed that docetaxel initially promoted considerable EGFR expression in PCa, and USP8 silencing suppressed the docetaxel-mediated increment of EGFR.

Interestingly, we also showed that the silenced EGFR-mediated NF-kB signal suppression was significantly attenuated by USP8 overexpression through stabilization of PI3K. Therefore, USP8 regulates NF-kB signal activation by not only stabilizing EGFR but also stabilizing PI3K. In docetaxel-resistant PCa cells, the chemoresistant action of EGFR is activated by ABCB1 expression *via* an Akt-dependent route (11), and it was also suggested that the activated PI3K/Akt pathway-mediated upregulation of multidrug resistance protein-1 (MRP-1; ABCC1) might promote the formation of chemoresistant cells in progressive PCa (23). As USP8 silencing significantly inhibited the PCa cell growth, proliferation, and metastasis and induced apoptosis and suppressed NF-kB signal activation by decreasing EGFR and PI3K, the USP8-specific inhibitor might be a novel therapeutic target to suppress PCa cell growth, proliferation, and metastasis. Moreover, the USP8 inhibitor combined with docetaxel treatment might also be effective in overcoming EGFR and PI3K/Akt-mediated docetaxel resistance in CRPC.

Our research findings are the first to show the role and molecular mechanism of USP8 in PCa cells involving docetaxel, EGFR, and PI3K-mediated NF-kB signaling pathway. These findings were in line with several previous studies showing that overexpression of USP8 played an important role in suppressing docetaxel antitumor ability and might be one of the reasons to develop docetaxel resistance through upregulating EGFR, PI3K, P-Akt, and NF-kB signaling pathway. These findings are shown mainly based on an *in vitro* experiment with docetaxel-sensitive PCa cell lines. Thereby, it is needed to carry on the experiment in docetaxel-resistant prostate cancer *in vitro* and *in vivo* to establish USP8 as a therapeutic target for not only prostate cancer but also docetaxel-resistant prostate cancer.

## Conclusion

By the above findings, the current study concluded that USP8 is a crucial regulator of the NF-kB signaling pathway. USP8 silencing significantly inhibits PCa cell growth, survival, and migration and promotes apoptosis by increasing cleaved Caspase 3 and cleaved Caspase 9. This study also shows that USP8 overexpression promotes PCa cell growth, survival, and migration and suppresses apoptosis. Together with these findings, it suggests USP8 as a considerable target for PCa therapeutics. Interestingly, our study also reports that USP8 silencing enhances docetaxel’s anticancer activity in PCa by suppressing EGFR and PI3K-mediated NF-kB signal activation. It also shows that USP8 overexpression suppresses docetaxel’s activity, thereby increasing EGFR and PI3K-mediated NF-kB signal activation. As the elevated EGFR, PI3K/Akt, and NF-kB activation is considered for the possible causes of developing chemoresistance/docetaxel resistance in cancer cells so USP8 silencing might also be a thinkable way to overcome docetaxel resistance for PCa. Therefore, it can state that USP8 may be considered a novel target for future experiments exploring elaborate mechanisms and interactions among the upstream and downstream proteins or signaling pathways involved in tumor growth, aggressiveness, and metastasis. Moreover, this *in vitro* study provides significant findings for a combination treatment of silenced USP8 and docetaxel in PCa therapeutics and to overcome docetaxel-mediated chemoresistance, which may guide to proceed future animal model experiments to establish USP8 as a clinically potential therapeutic agent for PCa.

## Data availability statement

The datasets presented in this study can be found in online repositories. The names of the repository/repositories and accession number(s) can be found in the article/**Supplementary Material**.

## Author contributions

MTI and H-CC designed the study and analyzed and interpreted the data. MTI conducted the experiments, analyzed the data, and drafted the manuscript. H-CC, F-ZC, and A.W reviewed the literature and revised the manuscript. All authors contributed to the article and approved the submitted version.

## Funding

This research was funded by the National Basic Research Program of China (Grant No. 2011CB910700‐ 704).

## Conflict of interest

The authors declare that the research was conducted in the absence of any commercial or financial relationships that could be construed as a potential conflict of interest.

## Publisher’s note

All claims expressed in this article are solely those of the authors and do not necessarily represent those of their affiliated organizations, or those of the publisher, the editors and the reviewers. Any product that may be evaluated in this article, or claim that may be made by its manufacturer, is not guaranteed or endorsed by the publisher.

## References

[1] Siegel RL Miller KD Fuchs HE Jemal A. Cancer statistics, 2021. CA Cancer J Clin (2021) 71:7–33. doi: 10.3322/CAAC.21654 33433946

[2] Feldman BJ Feldman D. The development of androgen-independent prostate cancer. Nat Rev Cancer (2001) 1:34–45. doi: 10.1038/35094009 11900250

[3] Lonergan P Tindall D. Androgen receptor signaling in prostate cancer development and progression. J Carcinog (2011) 10:1–12. doi: 10.4103/1477-3163.83937 PMC3162670 21886458

[4] Yap TA Zivi A Omlin A De Bono JS. The changing therapeutic landscape of castration-resistant prostate cancer. Nat Rev Clin Oncol (2011) 8:597–610. doi: 10.1038/nrclinonc.2011.117 21826082

[5] Eisenberger MA Walsh PC. Early androgen deprivation for prostate cancer? N Engl J Med (1999) 341:1837–8. doi: 10.1056/NEJM199912093412409 10588970

[6] Valero J Peleteiro P Henríquez I Conde A Piquer T Lozano A. Age, Gleason score, and PSA are important prognostic factors for survival in metastatic castration-resistant prostate cancer. results of the uroncor group (Uro-oncological tumors) of the Spanish society of radiation oncology (SEOR). Clin Transl Oncol (2020) 22:1378–89. doi: 10.1007/S12094-019-02274-W 31989474

[7] Scher HI Solo K Valant J Todd MB Mehra M. Prevalence of prostate cancer clinical states and mortality in the united states: Estimates using a dynamic progression model. PLoS One (2015) 10:e0139440. doi: 10.1371/JOURNAL.PONE.0139440 26460686 PMC4603789

[8] Tannock IF de Wit R Berry WR Horti J Pluzanska A Chi KN. Docetaxel plus prednisone or mitoxantrone plus prednisone for advanced prostate cancer. N Engl J Med (2009) 351:1502–12. doi: 10.1056/NEJMOA040720 15470213

[9] Marech I Vacca A Ranieri G. Novel strategies in the treatment of castration-resistant prostate cancer. Int J Oncol (2012) 40:1313–20. doi: 10.3892/ijo.2012.1364 22322981

[10] Seruga B Tannock IF. Chemotherapy-based treatment for castration-resistant prostate cancer. J Clin Oncol (2011) 29:3686–94. doi: 10.1200/JCO.2010.34.3996 21844499

[11] Hour TC Chung SD Kang WY Lin YC Chuang SJ Huang AM. EGFR mediates docetaxel resistance in human castration-resistant prostate cancer through the akt-dependent expression of ABCB1 (MDR1). Arch Toxicol (2015) 89:591–605. doi: 10.1007/S00204-014-1275-X 24888374

[12] Chandrasekar T Yang J Gao A Evans CP. Mechanisms of resistance in castration-resistant prostate cancer (CRPC). Transl Androl Urol (2015) 4:365–80. doi: 10.3978/j.issn.2223-4683.2015.05.02 PMC4708226 26814148

[13] Luo S Shao L Chen Z Hu D Jiang L Tang W. NPRL2 promotes docetaxel chemoresistance in castration resistant prostate cancer cells by regulating autophagy through the mTOR pathway. Exp Cell Res (2020) 390:111981. doi: 10.1016/J.YEXCR.2020.111981 32234375

[14] Tanguturi P Kim KS Ramakrishna S. The role of deubiquitinating enzymes in cancer drug resistance. Cancer Chemother Pharmacol (2020) 85:627–39. doi: 10.1007/S00280-020-04046-8 32146496

[15] Islam MT Zhou X Chen F Khan MA Fu J Chen H. Targeting the signalling pathways regulated by deubiquitinases for prostate cancer therapeutics. Cell Biochem Funct (2019) 37:304–19. doi: 10.1002/cbf.3401 31062387

[16] Byun S Lee SY Lee J Jeong CH Farrand L Lim S. USP8 is a novel target for overcoming gefitinib resistance in lung cancer. Clin Cancer Res (2013) 19:3894–904. doi: 10.1158/1078-0432.CCR-12-3696 PMC3891300 23748694

[17] Yan M Zhao C Wei N Wu X Cui J Xing Y. High expression of ubiquitin-specific protease 8 (USP8) is associated with poor prognosis in patients with cervical squamous cell carcinoma. Med Sci Monit (2018) 24:4934–43. doi: 10.12659/MSM.909235 PMC6067021 30010158

[18] Jing X Chen Y Chen Y Shi G Lv S Cheng N. Down-regulation of usp8 inhibits cholangiocarcinoma cell proliferation and invasion. Cancer Manag Res (2020) 12:2185–94. doi: 10.2147/CMAR.S234586 PMC7113805 32273758

[19] Shin S Kim K Kim HR Ylaya K Do SI Hewitt SM. Deubiquitylation and stabilization of Notch1 intracellular domain by ubiquitin-specific protease 8 enhance tumorigenesis in breast cancer. Cell Death Differ (2020) 27:1341–54. doi: 10.1038/s41418-019-0419-1 PMC7206187 31527799

[20] Sun J Hu Q Peng H Peng C Zhou L Lu J. The ubiquitin-specific protease USP8 deubiquitinates and stabilizes Cx43. J Biol Chem (2018) 293:8275–84. doi: 10.1074/jbc.RA117.001315 PMC5971442 29626091

[21] Zhu Y Xu J Hu W Wang F Zhou Y Gong W. Inhibiting USP8 overcomes hepatocellular carcinoma resistance *via* suppressing receptor tyrosine kinases. Aging (Albany NY) (2021) 13:14999. doi: 10.18632/AGING.203061 34081623 PMC8221339

[22] Pao W Miller VA Politi KA Riely GJ Somwar R Zakowski MF. Acquired resistance of lung adenocarcinomas to gefitinib or erlotinib is associated with a second mutation in the EGFR kinase domain. PloS Med (2005) 2:0225–35. doi: 10.1371/journal.pmed.0020073 PMC549606 15737014

[23] Lee JT Steelman LS McCubrey JA. Phosphatidylinositol 3′-kinase activation leads to multidrug resistance protein-1 expression and subsequent chemoresistance in advanced prostate cancer cells. Cancer Res (2004) 64:8397–404. doi: 10.1158/0008-5472.CAN-04-1612 15548710

[24] Meinel F Mandl-Weber S Baumann P Leban J Schmidmaier R. The novel NFkB inhibitor V1810 induces apoptosis and cell cycle arrest in multiple myeloma cells and overcomes NFkB mediated drug resistance. Blood (2008) 112:1715–5. doi: 10.1182/BLOOD.V112.11.1715.1715 20124446

[25] Mizuno E Iura T Mukai A Yoshimori T Kitamura N Komada M. Regulation of epidermal growth factor receptor down-regulation by UBPY-mediated deubiquitination at endosomes. Mol Biol Cell (2005) 16:5163–74. doi: 10.1091/mbc.E05-06-0560 PMC1266416 16120644

[26] Shen WM Yin JN Xu RJ Xu DF Zheng SY. Ubiquitin specific peptidase 49 inhibits non-small cell lung cancer cell growth by suppressing PI3K/AKT signaling. Kaohsiung J Med Sci (2019) 35:401–7. doi: 10.1002/KJM2.12073 PMC11900759 31001918

[27] Zhang Y Jia J Jin W Cao J Fu T Ma D. Lidocaine inhibits the proliferation and invasion of hepatocellular carcinoma by downregulating USP14 induced PI3K/Akt pathway. Pathol - Res Pract (2020) 216:152963. doi: 10.1016/J.PRP.2020.152963 32471606

[28] Man X Piao C Lin X Kong C Cui X Jiang Y. USP13 functions as a tumor suppressor by blocking the NF-kB-mediated PTEN downregulation in human bladder cancer. J Exp Clin Cancer Res (2019) 38:1–16. doi: 10.1186/S13046-019-1262-4/FIGURES/7 31200745 PMC6570860

[29] Islam T Chen F Chen H. The oncogenic role of ubiquitin specific peptidase (USP8) and its signaling pathways targeting for cancer therapeutics. Arch Biochem Biophys (2021) 701:108811. doi: 10.1016/j.abb.2021.108811 33600786

[30] Sun J Shen D Gao Y Zheng Y Zhao L Maa M. USP8 inhibitor suppresses HER-2 positive gastric cancer cell proliferation and metastasis *via* the PI3K/AKT signaling pathway. Onco Targets Ther (2020) 13:7973–84. doi: 10.2147/OTT.S264108

[31] Berlin I Schwartz H Nash PD. Regulation of epidermal growth factor receptor ubiquitination and trafficking by the USP8·STAM complex. J Biol Chem (2010) 285:34909–21. doi: 10.1074/jbc.M109.016287 PMC2966105 20736164

[32] Kasahara K Aoki H Kiyono T Wang S Kagiwada H Yuge M. EGF receptor kinase suppresses ciliogenesis through activation of USP8 deubiquitinase. Nat Commun (2018) 9:1–13. doi: 10.1038/s41467-018-03117-y 29472535 PMC5823934

[33] Mock A Plath M Moratin J Tapken MJ Jäger D Krauss J. EGFR and PI3K pathway activities might guide drug repurposing in HPV-negative head and neck cancers. Front Oncol (2021) 11:678966/BIBTEX. doi: 10.3389/FONC.2021.678966/BIBTEX 34178665 PMC8226088

[34] Kallergi G Agelaki S Kalykaki A Stournaras C Mavroudis D Georgoulias V. Phosphorylated EGFR and PI3K/Akt signaling kinases are expressed in circulating tumor cells of breast cancer patients. Breast Cancer Res (2008) 10:1–11. doi: 10.1186/BCR2149 PMC2614515 18822183

[35] Duan H Qu L Shou C. Activation of EGFR-PI3K-AKT signaling is required for mycoplasma hyorhinis-promoted gastric cancer cell migration. Cancer Cell Int (2014) 14:1–9. doi: 10.1186/S12935-014-0135-3/FIGURES/4 25505372 PMC4262230

[36] Kim Y Shiba-Ishii A Nakagawa T Husni RE Sakashita S Takeuchi T. Ubiquitin-specific protease 8 is a novel prognostic marker in early-stage lung adenocarcinoma. Pathol Int (2017) 67:292–301. doi: 10.1111/pin.12546 28544031

[37] Qiu H Kong J Cheng Y Li G. The expression of ubiquitin-specific peptidase 8 and its prognostic role in patients with breast cancer. J Cell Biochem (2018) 119:10051–8. doi: 10.1002/jcb.27337 30132974

[38] Liu Y Chen XG Liang CZ. Expressions of e-cadherin and n-cadherin in prostate cancer and their implications. Zhonghua nan ke xue=National J Androl (2014) 20:781–6. 25306803

[39] Gravdal K Halvorsen OJ Haukaas SA Akslen LA. A switch from e-cadherin to n-cadherin expression indicates epithelial to mesenchymal transition and is of strong and independent importance for the progress of prostate cancer. Clin Cancer Res (2007) 13:7003–11. doi: 10.1158/1078-0432.CCR-07-1263 18056176

[40] Liu GL Yang HJ Liu T Lin YZ. Expression and significance of e-cadherin, n-cadherin, transforming growth factor-β1 and twist in prostate cancer. Asian Pac J Trop Med (2014) 7:76–82. doi: 10.1016/S1995-7645(13)60196-0 24418088

[41] Jin R Sterling JA Edwards JR DeGraff DJ Lee C Park SI. Activation of NF-kappa b signaling promotes growth of prostate cancer cells in bone. PLoS One (2013) 8:e60983. doi: 10.1371/JOURNAL.PONE.0060983 23577181 PMC3618119

[42] Tantivejkul K Loberg RD Mawocha SC Day LL St. John L Pienta BA. PAR1-mediated NFκB activation promotes survival of prostate cancer cells through a bcl-xL-dependent mechanism. J Cell Biochem (2005) 96:641–52. doi: 10.1002/JCB.20533 16052512

[43] Jeong MS Lee KW Choi YJ Kim YG Hwang HH Lee SY. Synergistic antitumor activity of SH003 and docetaxel *via* EGFR signaling inhibition in non-small cell lung cancer. Int J Mol Sci (2021) 22:8405. doi: 10.3390/IJMS22168405 34445110 PMC8395077

[44] Wosikowski K Silverman JA Bishop P Mendelsohn J Bates SE. Reduced growth rate accompanied by aberrant epidermal growth factor signaling in drug resistant human breast cancer cells. Biochim Biophys Acta - Mol Cell Res (2000) 1497:215–26. doi: 10.1016/S0167-4889(00)00062-8 10903426

[45] Patel R Y Leung H. Targeting the EGFR-family for therapy: Biological challenges and clinical perspective. Curr Pharm Des (2012) 18:2672–9. doi: 10.2174/138161212800626148 22390755

[46] Chen T Xu J Fu W. EGFR/FOXO3A/LXR-α axis promotes prostate cancer proliferation and metastasis and dual-targeting LXR-α/EGFR shows synthetic lethality. Front Oncol (2020) 10:1688. doi: 10.3389/fonc.2020.01688 33224867 PMC7667376

[47] Jeong M Lee EW Seong D Seo J Kim JH Grootjans S. USP8 suppresses death receptor-mediated apoptosis by enhancing FLIP l stability. Oncogene (2017) 36:458–70. doi: 10.1038/onc.2016.215 27321185

[48] Hengartner MO. The biochemistry of apoptosis. Nat 2000 4076805 (2000) 407:770–6. doi: 10.1038/35037710 11048727

[49] Barton S Starling N Swanton C. Predictive molecular markers of response to epidermal growth factor Receptor(EGFR) family-targeted therapies. Curr Cancer Drug Targets (2010) 10:799–812. doi: 10.2174/156800910793357925 20718710

[50] Di Lorenzo G Tortora G D'Armiento FP De Rosa G Staibano S Autorino R. Expression of epidermal growth factor receptor correlates with disease relapse and progression to androgen-independence in human prostate cancer. Clin Cancer Res (2002) 8:3438–44. 12429632

[51] Marks RA Zhang S Montironi R McCarthy RP MacLennan GT Lopez-Beltran A. Epidermal growth factor receptor (EGFR) expression in prostatic adenocarcinoma after hormonal therapy: A fluorescence *in situ* hybridization and immunohistochemical analysis. Prostate (2008) 68:919–23. doi: 10.1002/PROS.20715 18409189

[52] Lin J Adam RM Santiestevan E Freeman MR. The phosphatidylinositol 3′-kinase pathway is a dominant growth factor-activated cell survival pathway in LNCaP human prostate carcinoma cells. Cancer Res (1999) 59:2891–7. 10383151

[53] Lin X Böhle AS Dohrmann P Leuschner I Schulz A Kremer B. Overexpression of phosphatidylinositol 3-kinase in human lung cancer. Langenbeck’s Arch Surg (2001) 386:293–301. doi: 10.1007/S004230100203 11466572

[54] Martínez-Lorenzo MJ Anel A Monleón I Sierra JJ Piñeiro A Naval J. Tyrosine phosphorylation of the p85 subunit of phosphatidylinositol 3-kinase correlates with high proliferation rates in sublines derived from the jurkat leukemia. Int J Biochem Cell Biol (2000) 32:435–45. doi: 10.1016/S1357-2725(99)00142-9 10762069

[55] Fry MJ. Phosphoinositide 3-kinase signalling in breast cancer: How big a role might it play? Breast Cancer Res (2001) 3:304–12. doi: 10.1186/BCR312/FIGURES/2 PMC138693 11597319

[56] Krasilnikov M Adler V Fuchs SY Dong Z Haimovitz-Friedman A Herlyn M. Contribution of phosphatidylinositol 3-kinase to radiation resistance in human melanoma cells. Mol Carcinog (1999) 24:64–9. doi: 10.1002/(SICI)1098-2744(199901)24:1<64::AID-MC9>3.0.CO;2-2 10029412

[57] Nicholson KM Anderson NG. The protein kinase B/Akt signalling pathway in human malignancy. Cell Signal (2002) 14:381–95. doi: 10.1016/S0898-6568(01)00271-6 11882383

[58] Shou J Massarweh S Osborne CK Wakeling AE Ali S Weiss H. Mechanisms of tamoxifen resistance: Increased estrogen receptor-HER2/neu cross-talk in ER/HER2–positive breast cancer. JNCI J Natl Cancer Inst (2004) 96:926–35. doi: 10.1093/JNCI/DJH166 15199112

[59] Yarden Y Sliwkowski MX. Untangling the ErbB signalling network. Nat Rev Mol Cell Biol (2001) 2:127–37. doi: 10.1038/35052073 11252954

[60] Dhomen NS Mariadason J Tebbutt N Scott AM. Therapeutic targeting of the epidermal growth factor receptor in human cancer. Crit Rev Oncog (2012) 17:31–50. doi: 10.1615/CRITREVONCOG.V17.I1.40 22471663

[61] Rosenzweig SA. Acquired resistance to drugs targeting receptor tyrosine kinases. Biochem Pharmacol (2012) 83:1041–8. doi: 10.1016/J.BCP.2011.12.025 PMC3299940 22227013

[62] Traish AM Morgentaler A. Epidermal growth factor receptor expression escapes androgen regulation in prostate cancer: a potential molecular switch for tumour growth. Br J Cancer (2009) 101:1949–56. doi: 10.1038/sj.bjc.6605376 PMC2795439 19888222

